# Supplementary material for: The contribution of vulnerability to emotional contagion to the expression of psychological distress in older adults
Source: PLOS Ment Health. 2024 Oct 29;1(5):e0000098. doi: 10.1371/journal.pmen.0000098 (PMC12798220; doi:10.1371/journal.pmen.0000098)
Supplement: S1 Appendix — (DOCX) [file pmen.0000098.s001.docx]

**S1 Appendix. Additional analysis on the depression and anxiety factors of the Hospital Anxiety and Depression Scale (HADS) as a continuous outcome**

**Analytic strategy**

A linear regression analysis was conducted on the depression and anxiety subscales of the HADS as continuous outcome measures. The same methodology as that employed in the primary analysis was utilized. Adversity was controlled for by including the perceived stress score. The use of psychotropic treatment was also included as a covariate in the model for each outcome. All predictors were added following a stepwise elimination method. The predictors included sociodemographic factors (sex, age, years of education, income, living situation), functional autonomy, availability and satisfaction with the social network, a range of coping styles, vulnerability to emotional contagion, and the cognitive and affective components of empathy.

**Results**

A total of five models were generated through stepwise linear regression on the depression subscale. The final model included a set of three factors in addition to the two covariates. The independent variables accounted for 43.8% of the variance in the depression subscale, and this was statistically significant (F (5,164) = 27.304, *p* < .001). The coefficients and confidence intervals are presented in Table S1 for both outcomes. Higher scores on the depression scale were significantly associated with lower functional autonomy, lower satisfaction with the social network, and less use of emotional support–seeking strategies.

Four models were obtained from the linear regression using a stepwise method on the anxiety subscale. In the final model, the independent variables accounted for 49.0% of the variance of the anxiety subscale, and this was statistically significant (*F* (6,163) = 28.012, *p* < .001). Higher scores on the anxiety scale were significantly associated with a higher income, higher scores on the vulnerability to emotional contagion and empathic distress scales, and lower satisfaction with one’s social network.

**Table A.** Results of stepwise backward linear regressions for depression and anxiety outcomes for the final models

| Variables | B | *p* | 95% CI | *ß* | *VIF* |
| --- | --- | --- | --- | --- | --- |
| *Depression* |  |  |  |  |  |
| Perceived stress | .311 | <.001 | .211, .411 | .411 | 1.345 |
| Psychotropic treatment | .213 | <.001 | .097, .328 | .219 | 1.090 |
| Functional autonomy | -.070 | .042 | -.137, -.003 | -.132 | 1.253 |
| Emotional support–seeking | -.159 | .005 | -.269, -.049 | -.174 | 1.115 |
| Satisfaction with the network | -.051 | .032 | -.097, -.005 | -.133 | 1.132 |
| *Anxiety* |  |  |  |  |  |
| Perceived stress | .396 | <.001 | .270, .522 | .403 | 1.386 |
| Psychotropic treatment | .122 | .095 | -.021, .265 | .097 | 1.095 |
| Income | .053 | .034 | .004, .102 | .120 | 1.045 |
| Vulnerability to emotional contagion | .288 | <.001 | .159, .417 | .263 | 1.181 |
| Satisfaction with the network | -.087 | .003 | -.144, -.029 | -.175 | 1.125 |
| Empathic distress | .081 | .017 | .015, .147 | .159 | 1.437 |

Note. VIF = Variance Inflation Factor. A VIF greater than 10 indicates that there is a cause of concern for multicollinearity.

**Discussion**

To supplement the primary analysis, we conducted linear regressions on the anxiety and depression subscales of the Hospital Anxiety and Depression Scale (HADS) to identify factors that could contribute to the observed variance on each scale. Each subscale model included a distinct set of factors, apart from one: satisfaction with the support received from the social network. This factor also influences the likelihood of exhibiting psychological distress profiles and is discussed in detail in the main paper.

No association was found between depression and anxiety and most sociodemographic factors, including age, gender, education, and living situation. As previously stated in the introduction, the relationship between sociodemographic factors and psychological distress exhibited some patterns, yet these patterns were not replicated across all studies. This is likely because older adults represent a highly diverse population. However, a higher income was found to be associated with a higher score on the anxiety scale. In our population, it is plausible that factors such as lifestyle expectations and financial responsibilities (toward family members or estate management) may contribute to anxiety in individuals with higher incomes.

In particular, the depression scale was found to be influenced by two additional factors: loss of functional autonomy and reduced tendency to seek emotional support. Firstly, many studies have demonstrated a correlation between elevated levels of functional dependence and psychological distress in older populations [1, 2]. In our study, it is noteworthy that despite a generally high level of functional autonomy, the variability in this factor still contributes to the depression observed in the sample. In this context, even a minor loss of functional autonomy appears to significantly impact one’s self-perception and outlook on the future. Secondly, the tendency to seek emotional support when confronted with adversity is inversely associated with depressive symptoms. This association was also significant in regard to the anxious depression profile in comparison to the anxious profile of the main analysis. As previously stated, this finding could be explained by several factors, including a discrepancy between the support available and the needs of the individuals, as well as a recall bias, particularly in the context of depressive symptoms.

With respect to the anxiety scale, our findings indicated that vulnerability to emotional contagion and empathic distress were significant predictors in our final model. A review of the overall study results suggests that vulnerability to emotional contagion, as a trait, is associated primarily with anxious symptomatology. In light of the theorical rationale pertaining to implicit stressors and the HHS axis, this association is consistent with the notion that anxiety experiences may arise from chronic responses to stress. Similarly, the propensity for empathic distress, as assessed by the Interpersonal Reactivity Index, demonstrated a robust correlation with anxiety symptoms. Individuals who reported greater discomfort in intense interpersonal settings exhibited elevated anxiety. This finding is noteworthy considering that the empathic distress subscale is frequently employed as a surrogate measure for the assessment of vulnerability to emotional contagion [3]. In a review, Decety and Lamm [4] posited that personal distress may be induced by imagining the self (putting oneself in someone else’s shoes) rather than focusing on another person’s feelings (imagining the other). This distinction has been interpreted in the context of cognitive neuroscience research, where it has been demonstrated that the act of taking the perspective of someone else activates the frontal lobe, particularly the executive functions associated with inhibitory control. This may serve a functional role in separating perspectives, enabling an individual to resist interference from their own perspective when taking the subjective perspective of another [5, 6]. In alignment with our findings, individuals who are more susceptible to empathic distress may experience greater difficulty in navigating shared self/other representations, leading to distress [4].

These findings are supplementary to those obtained in the primary analyses. They reinforce the stated implications regarding the key moderators of the individual’s trait susceptibility to emotional contagion in the older population and the effectiveness of psychological interventions aimed at teaching strategies for managing emotional contagion to prevent psychological distress in older adults. In future research, it would be interesting to evaluate the causal relationship between emotional contagion and psychological distress in the context of adversity.

**References**

1. Couture M, Lariviere N, Lefrancois R. Psychological distress in older adults with low functional independence: A multidimensional perspective. Arch Gerontol Geriatr. 2005;41(1):101-11. doi:10.1016/j.archger.2004.12.004.

2. Preville M, Potvin L, Boyer R. The structure of psychological distress. Psychol Rep. 1995;77(1):275-93. doi:10.2466/pr0.1995.77.1.275.

3. Singer T, Klimecki OM. Empathy and compassion. Curr Biol. 2014;24(18):R875-R8. doi:10.1016/j.cub.2014.06.054.

4. Decety J, Lamm C. Empathy versus personnal distress: Recent evidence from social neuroscience. In: Decety J, Ickes W, editors. The social neuroscience of empathy. Cambridge, MA: MIT Press; 2009. p. 199-213.

5. Decety J, Jackson PL. The functional architecture of human empathy. Behav Cogn Neurosci Rev. 2004;3(2):71-100. doi:10.1177/1534582304267187.

6. Ruby P, Decety J. What you believe versus what you think they believe: A neuroimaging study of conceptual perspective-taking. Eur J Neurosci. 2003;17(11):2475-80. doi:10.1046/j.1460-9568.2003.02673.x.
