# Supplementary material for: The contribution of vulnerability to emotional contagion to the expression of psychological distress in older adults
Source: PLOS Ment Health. 2024 Oct 29;1(5):e0000098. doi: 10.1371/journal.pmen.0000098 (PMC12798220; doi:10.1371/journal.pmen.0000098)
Supplement: Attachment — Submitted filename: Response to Reviewers.pdf [file pmen.0000098.s002.pdf]

July 22, 2024

Subject: Response to the reviewers regarding the submission of the article “The contribution of vulnerability to emotional contagion to the expression of psychological distress in older adults”

Dear editors and reviewers,

Above all, we would like to thank the journal for considering our manuscript for publication. We are especially appreciative of the insightful feedback from the reviewers, which has helped us improve the rigor of our manuscript.

Firstly, we would like to address the significant modification that was made to the analysis strategy. We acknowledge the concerns that have been raised and the limitations of the strategy that was originally employed. Although we were previously unacquainted with penalty-based (regularization) regression models, we recognize their value in improving model performance through regularization. Since the procedure was beyond the scope of our current analysis setup in the time frame offered, we were unable to secure the assistance necessary to ensure that the analysis would be performed adequately and interpreted with the requisite rigor. Consequently, we have employed the stepwise regression approach as initially suggested. While stepwise regression is a robust method for model selection and reducing multicollinearity, we acknowledge its limitations:

- Overfitting risk: Stepwise regression can lead to overfitting, particularly in the context of smaller datasets, as it relies heavily on the data at hand to determine which variables to include or exclude.
- Model stability: The selection of variables may vary significantly with different samples, making the model less stable and harder to generalize to other datasets.
- Exclusion of important variables: Important predictors may be excluded if they do not meet the stringent criteria at each step, potentially overlooking meaningful relationships in the data.

We added information about the multicollinearity of our predictors and discussed the results. The results obtained were comparable to those using the original strategies. In the course of revising the discussion, we accorded greater prominence to the finding of vulnerability to emotional contagion as one of the paper’s key results. Additionally, as recommended, we have included a linear regression analysis on the continuous scale of anxiety and depression as supplementary material.

The second major recommendation pertained to the context of the study. The following advised changes were duly implemented. We introduced sub-headings to better state the theorical components included, and we added a figure (Figure 1) to help the reader visualize the factors included. We posited more clearly the definition and theoretical relevance of vulnerability to emotional contagion, and we also edited down some content for greater clarity. Another comment about the theoretical components

concerned the expected relationships in the introduction and objectives section. We thus included some clarifications in the manuscript and specifically in the hypotheses.

Additionally, we revised the manuscript to address a few other minor comments:

- All labeling has been removed from the manuscript, in accordance with your helpful reminder.
- All $p < .000$ values have been changed to $p < .001$. Thank you for bringing this typographical error to our attention.
- We have incorporated an original reference for the O'Stress study (the doctoral dissertation of the first author), as well as the first publication of the initial program that was adapted by the first author.
- A reviewer highlighted the absence of certain details for some variables, including medical comorbid conditions. We acknowledge the value of comorbid conditions, and it would have been of great value in our model. However, we did not include this variable in our analysis and only briefly mentioned it in the context. To avoid confusion, we have removed this mention. Instead, we have added a mention of this important predictor in our discussion of study limitations and future work.
- After careful consideration, we decided to remove the link between proprioceptive sensitivity and vulnerability to emotion contagion and distress. This revision serves to maintain focus on the main mechanisms between vulnerability to emotional contagion and psychological distress that were added.
- As previously noted in our section on missing data, participants in the distress group were more likely to have incomplete data. This section does mention that the data from the three time points from the original study were imputed together. Therefore, the missing data were observed over the three time points, not only for the first one, which was used for the current study. Our sentence might have caused confusion. The incomplete data we refer to are data from all three points of measurement. The missing data from the first time point measurement specifically are completely at random.

As requested, we have included all the requested modifications in the tracked version of the manuscript. We hope that you will be satisfied with the changes that we have made. Please do not hesitate to contact us if you have any further questions about our work.

Sincerely,
Marie-Josée Richer, PhD

Pierrich Plusquellec, PhD
Associate Professor
